# Introducing EzBioCloud: a taxonomically united database of 16S rRNA gene sequences and whole-genome assemblies

**DOI:** 10.1099/ijsem.0.001755

**Published:** 2017-05-30

**Authors:** Seok-Hwan Yoon, Sung-Min Ha, Soonjae Kwon, Jeongmin Lim, Yeseul Kim, Hyungseok Seo, Jongsik Chun

**Affiliations:** Department of ChunLab, Inc, Seoul National University, Seoul, Republic of Korea

**Keywords:** 16S rRNA gene, genome, average nucleotide identity, identification, database

## Abstract

The recent advent of DNA sequencing technologies facilitates the use of genome sequencing data that provide means for more informative and precise classification and identification of members of the *Bacteria* and *Archaea*. Because the current species definition is based on the comparison of genome sequences between type and other strains in a given species, building a genome database with correct taxonomic information is of paramount need to enhance our efforts in exploring prokaryotic diversity and discovering novel species as well as for routine identifications. Here we introduce an integrated database, called EzBioCloud, that holds the taxonomic hierarchy of the *Bacteria* and *Archaea*, which is represented by quality-controlled 16S rRNA gene and genome sequences. Whole-genome assemblies in the NCBI Assembly Database were screened for low quality and subjected to a composite identification bioinformatics pipeline that employs gene-based searches followed by the calculation of average nucleotide identity. As a result, the database is made of 61 700 species/phylotypes, including 13 132 with validly published names, and 62 362 whole-genome assemblies that were identified taxonomically at the genus, species and subspecies levels. Genomic properties, such as genome size and DNA G+C content, and the occurrence in human microbiome data were calculated for each genus or higher taxa. This united database of taxonomy, 16S rRNA gene and genome sequences, with accompanying bioinformatics tools, should accelerate genome-based classification and identification of members of the *Bacteria* and *Archaea*. The database and related search tools are available at www.ezbiocloud.net/.

## Introduction

One of the goals of the modern taxonomy of the *Bacteria* and *Archaea* is the objective definition of species, insofar as it applies to classification and identification. The process of determining taxonomy has continually improved over time, with the advent of new technologies. PCR followed by sequencing of 16S rRNA genes has revolutionized our understanding of phylogeny of the *Bacteria* and *Archaea*. With the introduction of comprehensive 16S rRNA gene databases that cover almost all known species [1–4], the rate of discovering novel species was significantly improved. However, even though a bioinformatic comparison of 16S rRNA genes provides an objective and reliable way of identifying a given strain, it has a critical limitation in its use at the species level; even almost identical 16S rRNA gene sequences may not guarantee that two strains belong to the same species [5, 6]. To overcome this problem, an experimental approach called DNA–DNA hybridization has been used to complement 16S rRNA gene-based classification [7]. More recently, the use of genome data was recommended to replace error-prone, laborious DNA–DNA hybridization. Several overall genome relatedness indices (OGRIs) were proposed to define species boundaries [8]. For example, average nucleotide identity (ANI) [9] and OrthoANI [10] suggested a species boundary of 95–96 %.

Because genome sequences can be used for assessing suprageneric phylogeny, recognizing species [8] and differentiating clinical clones with few single nucleotide polymorphisms [11], it is evident that their use in the taxonomy of the *Bacteria* and *Archaea* will greatly improve not just taxonomy, but also other microbiological disciplines. As in the case of the 16S rRNA gene, the construction of a quality-controlled genome database of all type strains is a prerequisite for the wider application of genomics-based taxonomy [12].

At present, almost 70 000 genome sequences are available in the primary public databases, such as the NCBI Assembly Database (www.ncbi.nlm.nih.gov/assembly). Even though these genomes have great potential as a resource for basic, applied and clinical microbiology, their metadata such as taxonomic names require substantial curation. Here, we introduce an integrated database with a complete taxonomic hierarchy of the *Bacteria* and *Archaea* that is represented by 16S rRNA gene and genome sequences. All genomes were identified taxonomically at the genus, species or subspecies levels using a combination of gene-based search and OrthoANI [10] calculations. Integration of over 62 000 quality-filtered genomes allows us to generate comprehensive reports of DNA G+C content, genome sizes and other significant genomic features of each taxon. The database and related search tools are available at www.ezbiocloud.net/.

## Methods

### Data collection

The up-to-date reference 16S rRNA gene sequences were maintained as described earlier [3]. We attempted to select a sequence with the best quality for each species by using the following strategy. For cases in which multiple sequences were available for a type strain, the sequence extracted from its whole-genome assembly (WGA) was selected. As for PCR-derived sequences, the quality of sequencing was checked manually by secondary-structure-aware alignment using the EzEditor program [13]. Maximum-likelihood phylogenetic trees of each taxonomic group, such as phyla, classes, orders or families, were generated from manually aligned 16S rRNA gene sequences using RAxML software [14]. All 16S rRNA gene sequences were assigned taxonomically to the species level as a part of the complete taxonomic hierarchy which consisted of phylum, class, order, family, genus and species (subspecies if applicable).

### ‘Identify’ engine

Pairwise sequence similarity values between a query sequence and the reference sequences in our database are provided as an ‘Identify’ service. To ensure that the search engine finds the most similar sequence in the 16S rRNA gene sequence database, a two-step approach is employed [3]. Similar sequences are found first, then taxonomically meaningful pairwise sequence similarity values are calculated [15]. We employed the usearch program [16] instead of blastn in order to speed up the search process.

### Identification scheme of genome sequences

**Fig. 1.**
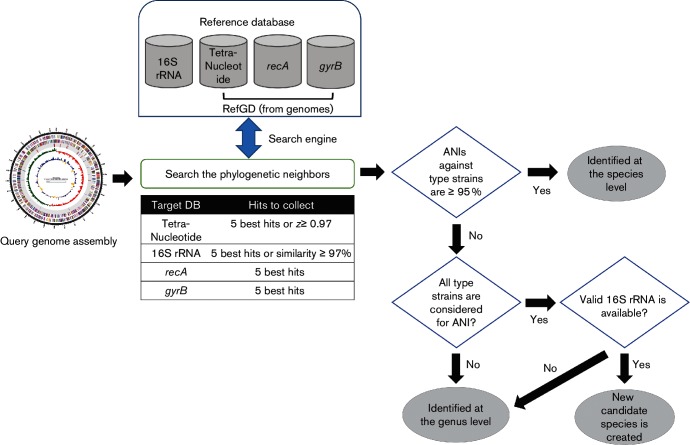
Schematic diagram of the algorithm for taxonomic identification of WGAs. The search engine used was the composite one described in detail in the text. Candidate novel species were added to the EzBioCloud database when a valid 16S rRNA gene sequence became available.

Taxonomic identification of each WGA was carried out using the algorithm outlined in Fig. 1. Prior to this, all WGAs were processed by a genome annotation pipeline using a combination of software tools and databases (Fig. S1, available in online Supplementry Material). Two types of databases were used, namely (i) the 16S rRNA gene sequence database that is also used in the ‘Identify’ engine described above, and (ii) the Reference Genome Database (RefGD). The latter was compiled to hold tetra-nucleotide compositions [17], and *gyrB* and *recA* sequences from all available genome sequences of type or representative strains. Tetra-nucleotide compositions were calculated from each WGA using an in-house java program. 16S rRNA, *gyrB* and *recA* genes in WGAs were predicted while sequences were processed in our genome annotation pipeline (Fig. S1). RefGD entries then served as the targets of usearch-based searches.

A list of taxa related phylogenetically to each WGA in the NCBI Assembly Database was generated using a combination of different approaches. The 16S rRNA, *gyrB* and *recA* gene sequences of a query WGA, wherever possible, were searched against the respective databases, and the best hits were added to the list. The correlation values (=*z* score) based on tetra-nucleotide composition were calculated against all WGAs in our RefGD [17], and the best hits were also added to the list. The final identification was carried out by comparing ANI values between the query WGA and those in the list for which we used 95 % as the cutoff for species delineation. For ANI calculation, we adopted the OrthoANI algorithm [10] with usearch instead of blastn to reduce the computation time. We attempted to identify all WGAs taxonomically at least to the genus level. If this was not possible due to the lack of 16S rRNA, *gyrB* and *recA* gene sequences, the WGA was assigned as ‘Unidentified’.

### Calculation of genomic features for each taxon

Several genomic features of taxonomic importance, including genome sizes, DNA G+C content, the number of genes and lengths of CDS (coding sequences)/intergenic regions, were calculated and compiled statistically for each taxon. The R package was used for all statistical analyses. Information on the number of 16S rRNA genes in genomes was obtained from complete genome sequences. If a species did not have any complete genomes, PICRUSt [18] was used to predict the values.

### Bacterial community analysis of human microbiome

The bacterial community dataset of the US NIH human microbiome project was obtained from hmpdacc.org/ and processed by using the bioinformatics pipeline given in Fig. S2. Frequencies of each taxon (from phyla to genera) at 18 body parts of healthy subjects were compiled and visualized as box-plots at the web-page for each taxon.

### Operating system and software development

The entire system was built on the standard Linux operating system and uploaded to Amazon Web Services (AWS) servers. java, JavaScript and R programming languages were used, and MySQL was used as the database management system.

## Results and discussion

### Hierarchical taxonomic backbone

The EzBioCloud database consists of a hierarchical taxonomic system containing 207 phyla, 433 classes, 1019 orders, 2805 families, 11 446 genera, 61 700 species and 387 subspecies. This classification was based primarily on the maximum-likelihood phylogeny for 16S rRNA gene sequence data, where 97 % similarity cutoff was used for the recognition of phylotypes. Taxa without their type or representative 16S rRNA gene sequences were not included in the database. We extended the database by adding novel candidate species that were identified by our identification scheme (Fig. 1) based on the combination of sequence-based search and OrthoANI calculations. As a result, 1168 tentatively named species were included in the database. Fig. S3 shows the OrthoANI-based dendrogram of the genus *Acinetobacter* in which 13 such novel candidate species are shown.

The taxonomic hierarchical system of EzBioCloud has the following principles: (i) all terminal taxa (species or subspecies) are represented by at least one 16S rRNA gene sequence, (ii) all terminal taxa are assigned under their complete suprageneric ranks (phylum, class, order, family), and (iii) taxonomic assignment is based on the phylogenetic relationship (maximum-likelihood treeing and OrthoANI), not necessarily following the current formal standing in taxonomy. For example, species of the genus *Shigella*is placed under the genus *Escherichia* but not the genus *Shigella* in our database, as it is phylogenetically a member of the former.

### Identification of genome projects

Our taxonomic search engine for WGA (Fig. 1) was designed to ensure that all possible phylogenetically neighbouring taxa are chosen for the final ANI calculations. The tetra-nucleotide composition of WGAs has been successfully applied to the rapid comparison between genomic and metagenomic assemblies [17, 19]. However, this is not a phylogenetic approach and is prone to be biased by large-scale lateral gene transfer. The 16S rRNA gene has been widely used for bacterial identification, and is ideal for finding phylogenetically related WGAs. However, out of 62 362 qualified WGAs, 4285 contain no 16S rRNA gene sequences that can be used for such a purpose. Therefore, two of the most widely used protein-coding phylogenetic markers, namely *gyrB* and *recA*, are implemented in our search engine in addition to the 16S rRNA gene and tetra-nucleotide composition. The genes coding for GyrB and RecA are also known to have higher resolution than the 16S rRNA gene in phylogenetic analyses [20, 21]. This composite approach allows the detection of all possible phylogenetically neighbouring taxa, which are then subjected to OrthoANI calculations.

**Fig. 2.**
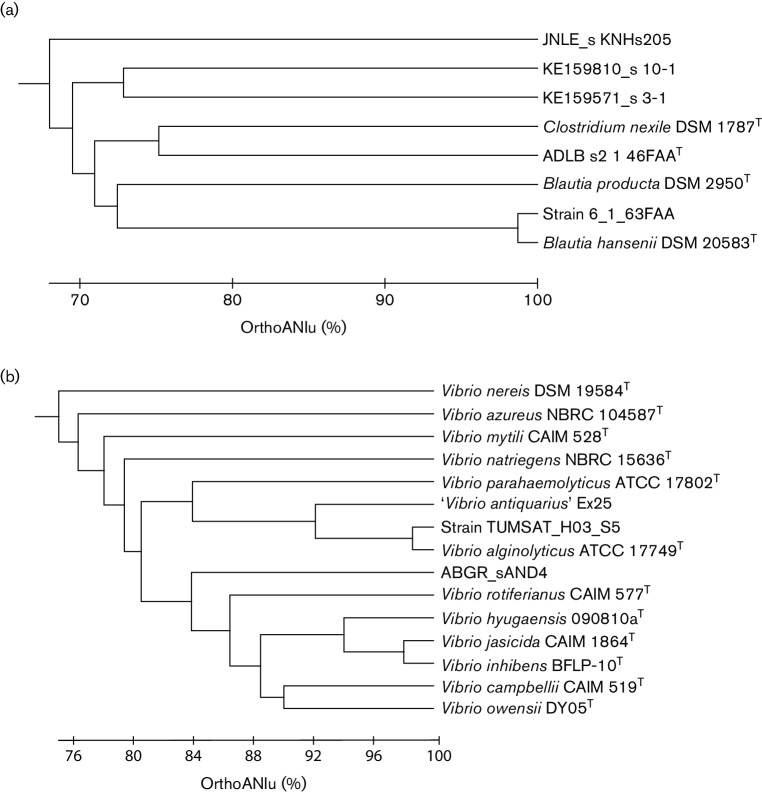
Examples of UPGMA dendrograms generated from a query WGA and reference genomes. (a) Strain 6_1_63FAA (NCBI Assembly accession GCF_000209425.1) is labelled as *Lachnospiraceae* bacterium 6_1_63FAA, but was identified as a strain of *Blautia hansenii* with an ANI value of 98.7 %. (b) Strain TUMSAT_H03_S5 (GCF_000591535.1) was originally deposited as a strain of *Vibrio parahaemolyticus*, but was identified as a strain of *Vibrio alginolyticus* with an OrthoANIu value of 98.6 %.

With 95 % ANI cutoff as species boundaries, 42 136, 15 794 and 4432 WGAs were identified at the species, subspecies and genus levels, respectively. Thirty-six WGAs could not be identified by the current version of RefGD. Also, the taxonomic names of 16 701 WGAs were found to be incorrect, which was supported by OrthoANI values. As a result, the taxonomic names of 16 737 WGAs (27 % of the total qualified WGAs) were changed from the original names in the NCBI Assembly Database that had been assigned originally by the primary data depositors. Examples of misidentified and unidentified WGAs are given in Fig. 2. We expect that the portion of WGAs identified at the species/subspecies level will be increased as more genome sequences become available for type strains.

### Genome-derived taxonomic information

The genome is the ultimate source for taxonomy in which a variety of information can be extracted for a better description of the species. For instance, more accurate G+C content of DNA can be obtained if calculated from genome sequences instead of experimental methods such as HPLC [22]. Because many species are now represented by multiple genomes, taxonomically meaningful information about species can be extracted and compiled statistically. In EzBioCloud, the following information is provided for each taxon, wherever applicable: (i) DNA G+C content, (ii) genome size, (iii) the number of CDSs, (iv) the length of CDSs and intergenic regions and (v) the number of 16S rRNA genes. An OrthoANI-based UPGMA dendrogram of type and reference strains is also provided for each genus if genome data is available. In addition, the occurrence of bacterial taxa, from phyla to genera, in 18 different body parts of the human microbiome is given as box-plot charts.

### Availability

The content of EzBioCloud’s hierarchical taxonomy, and 16S rRNA gene and genome sequence databases can be searched and browsed using HTML5-compatible web browsers at www.ezbiocloud.net/.

## Supplementary Data

394 Supplementary File 1
