# Supplementary material for: Introducing EzBioCloud: a taxonomically united database of 16S rRNA gene sequences and whole-genome assemblies
Source: Int J Syst Evol Microbiol. 2017 May 30;67(5):1613–7. doi: 10.1099/ijsem.0.001755 (PMC5563544; doi:10.1099/ijsem.0.001755)
Supplement: Supplementary File 1 [file ijsem-67-1613-s001.pdf]

Figure S1. Gene-finding and functional annotation pipeline of whole genome assembles used in EzBioCloud genome database. Protein-coding sequences (CDSs) were predicted by Prodigal 2.6.2 (Hyatt *et al.*, 2010). Genes coding for tRNA were searched using tRNAscan-SE 1.3.1 (Schattner *et al.*, 2005). The rRNA and other non-coding RNAs were searched by a covariance model search with Rfam 12.0 database (Nawrocki & Eddy, 2013). CRISPRs were detected by PilerCR 1.06 (Edgar, 2007) and CRT 1.2 (Bland *et al.*, 2007). The CDSs were classified into groups based on their roles, with reference to orthologous groups (EggNOG 4.5; http://eggnogdb.embl.de) (Powell *et al.*, 2014). For more functional annotation, the predicted CDSs were compared with Swissprot (UniProt, 2015), KEGG (Kanehisa *et al.*, 2014) and SEED (Overbeek *et al.*, 2005) databases using UBLAST program (Edgar, 2010).

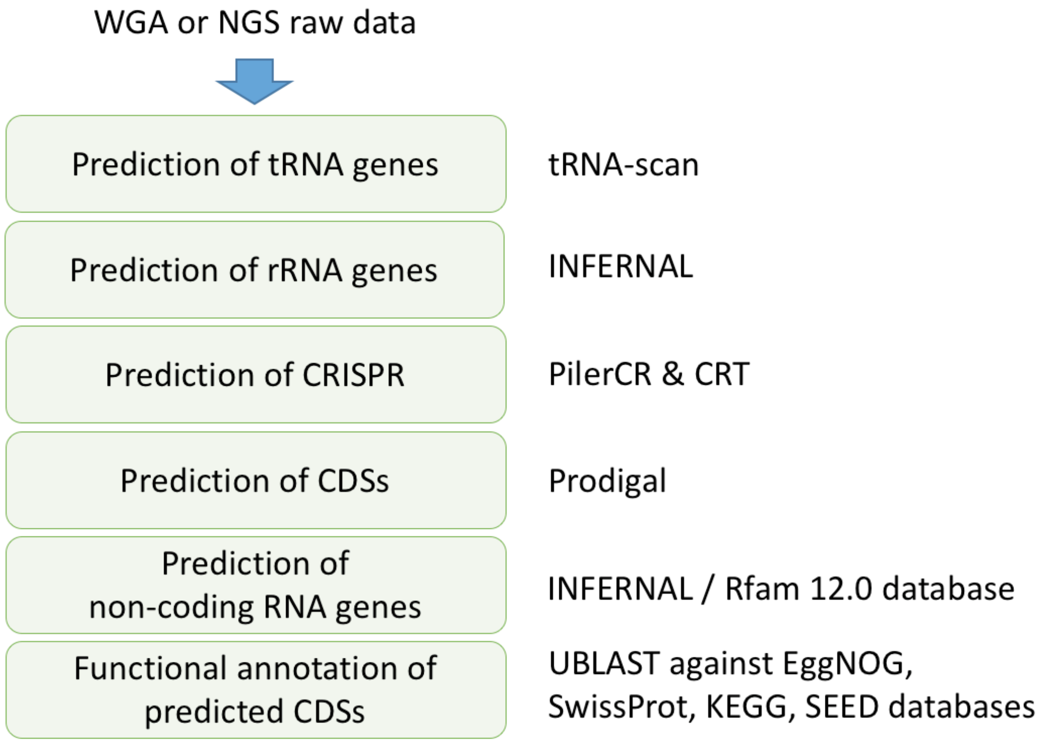

Figure S2. Bioinformatics pipeline for bacterial community analysis. The primer sequences were discarded using in-house JAVA program. Non-specific amplicons that do not encode 16S rRNA are detected by HMMER's hmmsearch program (Eddy, 2011) with 16S rRNA profiles. All sequences were denoised by DUDE-Seq (http://data.snu.ac.kr/pub/dude-seq/) and non-redundant reads were extracted by UCLUST-clustering (Edgar, 2010). Taxonomic identification was assigned against the EzBioCloud database using USEARCH (8.1.1861_i86linux32) (Edgar, 2010) followed by more precise pairwise alignment (Myers & Miller, 1988). The chimera sequences were detected by UCHIME (Edgar *et al.*, 2011). Only sequencing reads with lower than 97% similarity to EzBioCloud database were considered for chimera detection. Operational taxonomic units (OTUs) in the sample were investigated using open-reference method (Rideout *et al.*, 2014) with CD-HIT (Fu *et al.*, 2012) and UCLUST (Edgar, 2010). The alpha diversity indices and rarefaction curves were estimated by in-house code.

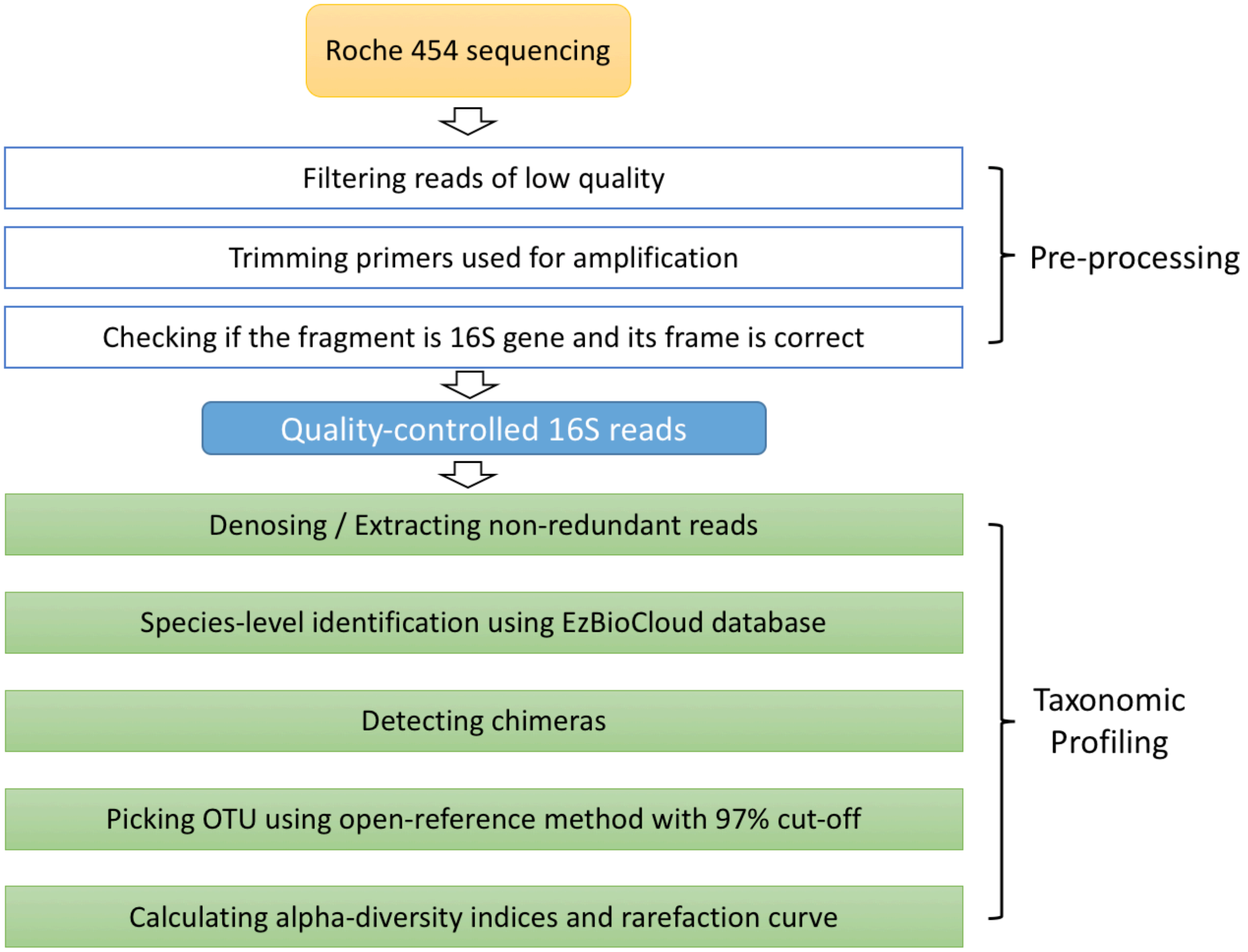

Figure S3. OrthoANI-based dendrogram of the genus *Acinetobacter* including 13 tentatively named species. The dendrogram is constructed using UPGMA algorithm. The scale bar represents OrthoANI values. <Type>, type strain.

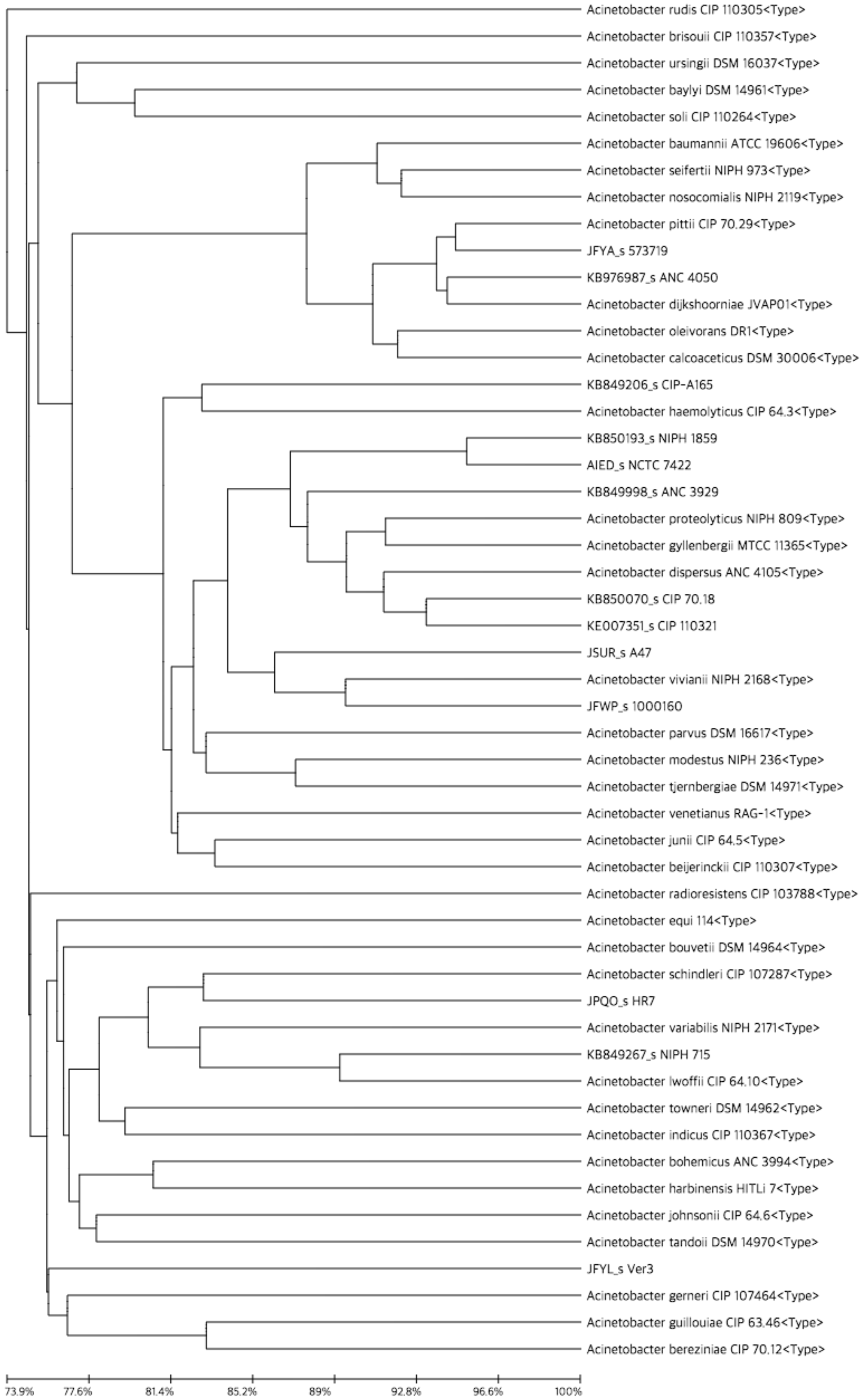

References

Bland, C., Ramsey, T. L., Sabree, F., Lowe, M., Brown, K., Kyrpides, N. C. & Hugenholtz, P. (2007). CRISPR recognition tool (CRT): a tool for automatic detection of clustered regularly interspaced palindromic repeats. *BMC Bioinformatics* **8**, 209.

Eddy, S. R. (2011). Accelerated profile HMM searches. *PLoS Comput Biol* **7**, e1002195.

Edgar, R. C. (2007). PILER-CR: fast and accurate identification of CRISPR repeats. *BMC Bioinformatics* **8**, 18.

Edgar, R. C. (2010). Search and clustering orders of magnitude faster than BLAST. *Bioinformatics* **26**, 2460-2461.

Edgar, R. C., Haas, B. J., Clemente, J. C., Quince, C. & Knight, R. (2011). UCHIME improves sensitivity and speed of chimera detection. *Bioinformatics* **27**, 2194-2200.

Fu, L., Niu, B., Zhu, Z., Wu, S. & Li, W. (2012). CD-HIT: accelerated for clustering the next-generation sequencing data. *Bioinformatics* **28**, 3150-3152.

Hyatt, D., Chen, G. L., Locascio, P. F., Land, M. L., Larimer, F. W. & Hauser, L. J. (2010). Prodigal: prokaryotic gene recognition and translation initiation site identification. *BMC Bioinformatics* **11**, 119.

Kanehisa, M., Goto, S., Sato, Y., Kawashima, M., Furumichi, M. & Tanabe, M. (2014). Data, information, knowledge and principle: back to metabolism in KEGG. *Nucleic Acids Res* **42**, D199-205.

Myers, E. W. & Miller, W. (1988). Optimal alignments in linear space. *Comput Appl Biosci* **4**, 11-17.

Nawrocki, E. P. & Eddy, S. R. (2013). Infernal 1.1: 100-fold faster RNA homology searches. *Bioinformatics* **29**, 2933-2935.

Overbeek, R., Begley, T., Butler, R. M., Choudhuri, J. V., Chuang, H. Y., Cohoon, M., de Crecy-Lagard, V., Diaz, N., Disz, T.& other authors (2005). The subsystems approach to genome annotation and its use in the project to annotate 1000 genomes. *Nucleic Acids Res* **33**, 5691-5702.

Powell, S., Forslund, K., Szklarczyk, D., Trachana, K., Roth, A., Huerta-Cepas, J., Gabaldon, T., Rattei, T., Creevey, C.& other authors (2014). eggNOG v4.0: nested orthology inference across 3686 organisms. *Nucleic Acids Res* **42**, D231-239.

Rideout, J. R., He, Y., Navas-Molina, J. A., Walters, W. A., Ursell, L. K., Gibbons, S. M., Chase, J., McDonald, D., Gonzalez, A. & Robbins-Pianka, A. (2014). Subsampled open-reference clustering creates consistent, comprehensive OTU definitions and scales to billions of sequences. *PeerJ* **2**, e545.

Schattner, P., Brooks, A. N. & Lowe, T. M. (2005). The tRNAscan-SE, snoscan and snoGPS web servers for the detection of tRNAs and snoRNAs. *Nucleic Acids Res* **33**, W686-689.

UniProt Consortium (2015). UniProt: a hub for protein information. *Nucleic Acids Res* **43**, D204-212.
